# Implementing and evaluating a primary care service for oral surgery: a case study

**DOI:** 10.1186/s12913-018-3420-3

**Published:** 2018-08-14

**Authors:** Joanna Goldthorpe, Caroline Sanders, Lesley Gough, Jean Rogers, Colette Bridgman, Martin Tickle, Iain Pretty

**Affiliations:** 1 Manchester Centre for Health Psychology, Division of Psychology and Mental Health, University of Manchester, Coupland 1 Building, Manchester, M13 9PL UK; 2 Division of Population Health, Health Services Research and Primary Care, University of Manchester, Williamson Building, Manchester, M13 9PL UK; 3 Public Health England, Cheshire and Merseyside PHE Centre 5th Floor, Rail House, Lord Nelson Street, Liverpool, L1 1JF UK; 4 NHS England North, Regatta Place, Brunswick Business Park, Summers Road, Liverpool, L3 4BL UK; 5 Primary Care Division, Directorate of Primary Care and Innovation, Health and Social Services Group, Welsh Government, Cardiff, UK; 6 Division of Dentistry, University of Manchester, J R Moore Building, Manchester, M13 9PL UK; 7 Colgate Palmolive - University of Manchester Dental Health Unit, Williams House, Manchester Science Park, M15 6SE UK

**Keywords:** Oral surgery, Primary health care, Referral management, Organisational case studies, Patient satisfaction, Demand management

## Abstract

**Background:**

A primary care oral surgery service was commissioned alongside an electronic referral management system in England, in response to rising demand for Oral Surgery services in secondary care. It is important to ensure that standards of quality and safety are similar to those in existing secondary care services, and that the new service is acceptable to stakeholders. The aim of this study is therefore to conduct an in depth case study to explore safety, quality, acceptability and implementation of the new service.

**Methods:**

This case study draws on multiple sources of evidence to report on the commissioning process, implementation, treatment outcomes and acceptability to patients relating to a new oral surgery service in a primary care setting. A combination of audit data and interviews were analysed**.**

**Results:**

Most referrals to the new service consisted of tooth extractions of appropriate complexity for the service. There were issues with lack of awareness of the new service in a primary care setting within referring primary care practices and patients at the start of implementation, however over time the service became a fully integrated part of the service landscape. Complications reported following surgery were low.

**Conclusion:**

Patients liked the convenience of the new service in terms of shorter waiting time and geographical location and their patient reported experience measures and outcomes were similar to those reported in secondary care. Providing appropriate clinical governance was in place, oral surgery could safely be provided in a primary care setting for patients without complex medical needs. Attention needs to be paid to communication with general dental practices around changes to the service pathway during the early implementation period to ensure all patients can receive care in the most appropriate setting.

## Background

Referral management has the potential to improve access to in-demand specialties by transferring suitable services from hospitals to primary care and community settings [1]. Typically, general practitioners (GPs) provide these services with recognised training in a specific medical specialism or specialists undertake treatment for routine procedures in primary care settings. Studies of new GPs with specialist interest (GPwSI) primary care based services have demonstrated potential effectiveness for a number of outcomes, including reducing overall costs [2], reducing waiting times and increasing patient satisfaction [3, 4].

The UK National Health Service (NHS) has contemporaneously produced local guidance for implementing referral management interventions [5] showing support for this method of demand management within secondary care and is seen as an opportunity for the augmented role of primary care in providing specialist or advanced services and reducing pressure on outpatient services [6, 7]. Despite support for referral management interventions to reduce the demand on secondary care in an efficient manner, caution has been urged around promoting the increase in referral management centers without evidence on the impact of their use.

Davies and Elwyn [8] expressed concern that referral management centres have potential to become mere tools for management to produce efficiency savings that could override patient safety, clinical autonomy and confidentiality. In addition, concerns have been voiced that the introduction of additional primary care services may stimulate demand locally rather than have the effect of reducing the burden on secondary care [9]. The loss of economies of scale associated with a move away from larger secondary care settings may reduce the cost effectiveness of advanced primary care and outreach services when compared to acute hospitals [10], however there may be advantages for patients in terms of improved access and satisfaction with services.

Oral Surgery referrals represent the largest volume and cost of referrals from primary dental care to secondary care [11]. Oral surgery referrals are mainly for tooth extractions that may present with surgical or medical complications and may also be supplemented by a request for sedation or general anaesthetic due to patient anxiety and/or the complexity of procedure required [12]. Care is typically provided in hospital outpatient clinics and is consultant-led with trainees at various levels undertaking procedures. Figures from acute trusts and electronic reporting data suggest an exponential increase in referrals for oral surgery to secondary care over recent years with subsequent pressure being placed on hospitals to deliver treatment in a timely, safe and cost-effective manner [13]. Several reasons have been put forward to explain this increase in demand: the 2006 UK NHS Dental Contract may provide unintentional financial incentives for General Dental Practitioners (GDPs) to refer patients and newly qualified dentists may lack the experience to confidently undertake routine oral surgery procedure [14].

In response to this increased demand for oral surgery in the NHS, a pilot referral management system was commissioned under the Dental Quality, Innovation, Productivity and Prevention (QuIPP) programme developed by the UK Department of Health [15]. It incorporated an electronic referral management system and an accompanying primary care-based oral surgery service to deliver advanced primary care treatments. The aim of this pilot was to reduce the number of oral surgery referrals into secondary care by selecting cases that could be safely treated in a primary care setting by dentists with additional skills [16]. Such dentists were supported by access to Consultant advice and supervision. This scheme was therefore consistent with the NHS England Oral Surgery Commissioning Guide [17], that advocates wider use of primary care settings for advanced or specialist led care underpinned by triage of case complexity using referral management systems.

In addition, a service specification was developed following the health needs assessment phase of the work that provided details on the volume, complexity and type of treatments required. The main findings of the health needs assessment suggested that of the 670 referrals for oral surgery, only 2.5% had the lowest levels of complexity (level 1, suitable for treatment by a general dental practitioner) while a total of 67.2% were deemed appropriate for level 2 primary care services. Of those referrals triaged to level 3 (Consultant-led services), medical complications were highlighted as the main reason. In addition to the numbers of referrals in each level of case complexity the health needs assessment also provided information on co-morbidities, age, sex and socio economic status based on postcode of the referred patients. Combined with information on the treatment required this provided a rich data set from which the service specification could be derived.

Following the commissioning process, a single provider was selected; a primary care dental practitioner with a Dentist with Specialist Interest (DWSpI) contract, who was also principal of an established medium-sized general dental practice. A second site for the service was secured in a modern, corporate-owned private practice that met the required quality standards and had suitable facilities available for rent.

The primary care service was designed to be embedded within a referral management pathway, commissioned based on a robust health needs assessment and assessed for quality and patient satisfaction. As such this report differs from other service evaluations that have often been based on legacy services that have grown organically rather than as part of an integrated forward plan. Moreover, the intervention at the center of this research is part of a wider commissioning strategy across a specific geographical area, rather than a researcher-led intervention being tested in an artificial situation without plans to establish longevity. Observational intervention implementation studies of this type offer opportunities to develop insights into barriers, facilitators, and key influences on routine implementation processes and issues around upscaling and industrialization across sites [18]. Implementation research considers how research and evidence is translated into practice in the form of interventions in “real world” settings and how this process might be improved. It considers factors affecting implementation, processes of implementation, and results of implementation and (particularly relevant to this case study) how to promote their large scale use and sustainability [19].

This case study is part of a wider body of research which aimed to explore issues associated with the commissioning, procurement, costs and implementation of an advanced referral management and primary care service for oral surgery [16]. It explores a number of factors relevant to the implementation of interventions in healthcare identified by Mittman [18]: the patient-clinician dyad; clinical microsystems and their relation to larger organizations; professional communities and relationship to regional and national policy. It is important to ensure that standards of quality and safety in the new primary care-based service are similar to those in existing secondary care services, and that the new service is acceptable to stakeholders. The aim of this study is therefore to evaluate this new primary care service and explore issues around implementation, with a focus on safety, quality and acceptability.

## Methods

This study was granted approval by NHS Research Ethics Committee (Grampian) Reference 13/NS/0141. A nested case study was conducted as part of a larger mixed methods research project focusing on implementation and acceptability of the advanced primary care service [16]. A case study research strategy aims to understand the dynamics present within an organizational setting [20]. This is a holistic [21] case study, in that it looks at only one unit of analysis; setting up and implementing the advanced primary care service in a specific geographical area. It is also intrinsic in design, in that it aims to gain a better understanding of a specific service, rather than a wider phenomenon [22].

The approach used to synthesizing data that forms the basis of this case study is closest to that described by Yin [23] in that it uses data from both quantitative and qualitative sources and attempts to answer “how” or “why” questions concerning the “phenomenon of interest” (the primary care oral surgery service). Using a mixed-methods approach can provide increased confidence in findings through a methods triangulation approach where results are interrogated to see if they are convergent. If this is the case greater confidence may be placed in the overall findings [24]. The research context for this case study was also in line with this approach as the researcher had little control over the intervention itself or its boundaries and parameters [25].

Table 1 Shows that the data collection period for this case study occurred in years three and four of the overall study, following a diagnostic test accuracy study for the electronic referral management and triage system in year 1 and 12 months of virtual implementation for a health needs assessment to inform commissioning of the primary care service in year 2. Please see Goldthorpe et al. (2018) for a thorough report of the overall study.

**Table 1:** How the case study sits within the wider body of research

DTA phase year 1	Diagnostic test accuracy study of electronic referral management and triage
Interrupted time series study year 2	Virtual implementation with triage for health needs assessment and no primary care service
	Health needs assessment (triage data from referral management centre)
Interrupted time series study year 3	Full implementation with consultant triage and diversion to tier 2 service Data collected included in this case study
Interrupted time series study year 4	Full implementation with consultant triage, GDP autonomous decision making and diversion to tier 2 service Data collected included in this case study

### Data collection

Data were drawn from three sources, congruent with a mixed methods case study approach that allows for triangulation of evidence [23, 24]. Data was collected in 2015 and 2016, years 3 and 4 of the study.

#### Service audit data

Provided directly from data collected and held by the primary care service was analyzed using Microsoft Excel (Microsoft, Seattle, WA, USA). Data collected covered a 16-month period of operation for the service. Data collected included type of appointment, treatment or procedure carried out and any complications that may have arisen. This was collected to ensure the new primary care service was able to treat patients efficiently and that their conditions represented an appropriate level of case complexity.

#### Patient records

Informed consent was sought from all patients and records were examined for any complications reported to General Dental Practitioners and treatment providers (hospitals or primary care oral surgery service) by patients within 6 weeks of receiving treatment at the new service to explore levels of patient safety. 6 wks was chosen as an appropriate time period based on the advice of a consultant oral surgeon in relation to healing time appropriate for non- complex tooth extractions.

#### Qualitative interviews

Were conducted with several relevant stakeholders during the first year of live implementation: the surgeon holding the primary care contract, commissioners, secondary care staff and service users as part of the wider study. Interviews were carried out by an experienced researcher, either over the telephone, face to face or in a dental practice or office. Topics for discussion were identified through reviewing relevant literature and discussion with the research team and informed by Normalisation Process Theory (NPT) [26]. All interviews were audio-recorded and transcribed, and transcripts were made anonymous and checked for accuracy.

### Data analysis

#### Service audit data

Spreadsheet columns were sorted and data was counted in order to carry out a basic descriptive analysis of patients’ treatment outcomes.

#### Patient records

Electronic or paper records were searched for patients attending their general dental practice or treatment provider 6 weeks or earlier post treatment and any complications were noted by a member of staff or the research team on a data collection form designed by a consultant in Dental Public Health to capture key clinical outcomes.

#### Qualitative interviews

A total of 29 interviews were carried out with 19 primary care staff from general dental practices over the course of the study. We aimed to interview staff from practices with varying levels of engagement with the system within the first 3 months of implementation, then once again at varying points in the remaining study period. The practitioner delivering the primary care service (DWSpI) took part in one interview. Nine interviews were carried out with seven secondary care staff and two commissioners took part in two interviews each. All practitioner interviews were carried out either over the telephone or face to face at the participant’s place of work.

A purposive sample (chosen to represent attendance at each of the primary and secondary care clinics) of 30 patients took part in telephone interviews and a convenience sample of 28 practitioners took part in either a face to face or telephone interview. One recording proved to be intelligible and the recorder failed one interview, leaving a total of 28 interview transcriptions in the analysis. All interviews typically lasted 30–45 min. All patient participants were adults aged between 23 and 80 years, with an equal number of men and women participating. 18 professional participants were female and 10 were male and all were aged 18 to 65. Interviews were carried out in relation to the patient journey from initial consultation to referral and treatment and full description of the interviews and study sample, including demographic information is available (Goldthorpe et al., 2018, appendix 2, pages 105–106). Selected quotes from patients and practitioners included in this larger sample are included to illustrate key findings relating specifically to the primary care oral surgery service.

### Analysis of qualitative data

Thematic approach to analysis drew upon some common techniques of grounded theory approaches after Glaser and Strauss [27] including constant comparison (analysis was conducted concurrently with data collection so that emerging issues could be explored iteratively). The aim of the grounded theory approach is to generate new theory or expand existing theory through an in depth exploration of the data. Although the analysis explored lived events as described by participants rather than generated theory, techniques used in a grounded theory approach can be transferred to thematic analysis to ensure a deductive approach is taken (that findings are grounded in the data rather than in researchers’ preconceptions). Stages of coding comprised initial coding of text segments, followed by re-coding and memo writing to generate conceptual themes. Themes were compared within and across cases, paying attention to negative cases and possible reasons for differences. The generation of themes was both inductive and deductive in drawing upon existing core constructs from NPT, whilst also allowing new themes to emerge from an initial process of open coding. Data were organised with the aid of qualitative data software package QSR NVivo (QSR International, London, UK). An audit trail of all stages of the analysis to maximise credibility, dependability, confirmability and transferability [28, 29] was created through sharing and saving of NVIVO files at various stages of the analysis, recording group decision making and archiving emails relating to the analytic process. Three of the authors (JG, CS and IP) discussed emerging themes to enable refinement of conceptual categories and to discuss common threads or differences across the different respondent groups too ensure reliability. Any discordance in the identification of themes and codes was resolved through discussion. Further reliability was established through triangulating findings, study documents and qualitative data.

### Synthesis of data sources

The results from each source were cross checked for convergence and contradictions. For example, the number of treatment failures found in patient records, would be cross checked for numbers of onward referrals to secondary care and patients qualitative reports of satisfaction with treatment (high numbers of treatment failures in the primary care service would be associated with a similar number of onward referrals, which would in turn be expected to be associated with low patient satisfaction with the service).

## Results

**Fig. 1:**
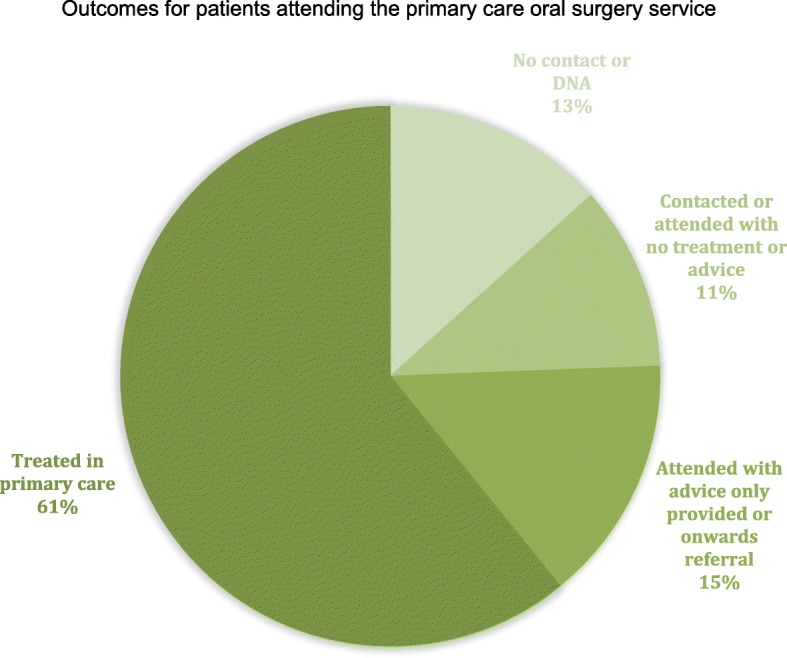
Outcomes for patients attending the primary care oral surgery service

The new primary care oral surgery service was introduced in an area of England with a high number of referrals for oral surgery to secondary care, but without current referral management in place. There were three types of hospital receiving referrals in this geographical area: a large foundation trust hospital (NHS direct foundation trust document), a small district general hospital and a university teaching hospital Fig 1.

### Service audit data

Table 1 shows the outcomes of each appointment for patients attending at the advanced primary care service between May 2015 and September 2016 (*n* = 623). The majority of appointments (61%) related to tooth extractions. 13% of patients could not be contacted to make an appoinment, or did not attend appointments made. 26% of patients had appointments which did not result in treatment, with 15% of patients needing a further referral or care advice and 11% receiving no treatment, advice or onward referral.

As the primary care service had been commissioned to assess and treat patients on the same day, it is important to explore reasons for the unexpected numbers of consultation-only appointments and to ensure that patients who received a consultation without treatment had been referred appropriately. The main reason given for patients receiving no treatment, advice or onward referral was patients reporting that they were now asymptomatic (75%). Of the 11% assessed and referred onwards, 4 patients requested to be sent to hospital to have their treatment and 10 appointments were deemed unsuitable for treatment in the advanced primary care service by the assessing clinician. A further 8 assessments resulted in patients refusing any treatment. For three assessments, inaccurate information on the referral had been entered by the referring clinician, resulting in a full re- examination and 3 appointments were for reviews of previously completed treatment. Two patients requested second opinions before embarking on treatment. Two assessments resulted in patients referred on to secondary care, specifically because the site of extraction (typically for lower wisdom teeth) was near the ID nerve and hence the surgical complexity was beyond that of the Tier 2 service.

### Complications following treatment (reported in patient records)

Table 2 shows the number and type of complications reported by patients following treatment. As the Primary Care service carried out tooth extractions exclusively, it is important to note that some complications followed treatment which could only be provided in a hospital setting. Therefore Table 2 separates the number of complications for tooth extractions and the number of complications resulting from other types of treatment.

**Table 2:** Treatment complications from study settings

Treatment provider	No of consenting patients	Complications following tooth extractions	Complications following other treatment
Dental Hospital	3	0	0
Foundation Trust	9	1	1
District General	69	2	1
Primary Care Oral Surgery	97	4	0

178 patients consented to have their records examined for complications following treatment. Of those, 3 patients from the Dental Hospital consented to have their records examined with no complications arising, 9 patients from the Foundation Trust consented with one patient experiencing complications following tooth extraction (exposed bone at the extraction site) and one for unsuccessful physiotherapy treatment for Tempero Mandibular Joint (jaw) pain. Sixty nine patients from the District General consented with two complications arising following tooth extraction (dry socket at extraction site for one case and antibiotics needed for infection at extraction site respectively) one case of a failed apicetomy (advanced root canal treatment) reported. Of ninety seven patients attending the Primary Care Oral Surgery Service, four complications arose following treatment for tooth extraction; two patients requested after care advice, one case of dry socket at extraction site was reported and one patient was given a course of antibiotics to aid healing.

### Interview data

An analysis of the data revealed themes consistent with 3 of the four NPT domains: 1) Coherence, 2) Collective action and 3) Reflexive monitoring, which informed an understanding of the sense making, operational work and appraisal participants undertook when engaging with the advanced primary care service. *Understanding new services and pathways* drew on the construct of coherence, *collective action between providers* and *clinical governance* drew from the collective action construct and *comparison with other treatment experience* was informed by reflexive monitoring.

### Understanding new services and pathways

A barrier to coherence about the new services was a lack of information given to patients about their possible treatment destination. During the first year of implementation patients expected to be treated either by their GDPs in their regular practice or a hospital setting according to traditional treatment pathways. As the evaluation moved into its second active year, with the introduction of GDP decision making, patients reported less confusion around their referral and subsequent treatment setting.

Differentiation between sets of working practices (e.g. paper letters to consultants and electronic referral management) is important for developing a coherent understanding of complex interventions (May et al. 2009). Particularly in the early months of the service becoming operational, patients were not well informed about new treatment pathways and could be driven to carry out investigative work to chase their referral and subsequently check on the authenticity of the new advanced service. This was carried out within the context of several drivers to see treatment completed in a timely manner combined with lack of information about pathways and ultimate treatment provider. Confusion was perhaps exacerbated by the delay in the advanced primary care service becoming operational. This description by a patient of undertaking several processes in order to obtain information about their referral was not uncommon in the early days of the advanced service becoming active:

*“I think I waited about three months, and nothing came through. So I went back to the dentist and they said, “oh we’ll check up on it”. In the meantime, I rang [hospital 1] and they had no knowledge of me. So they said, try [Hospital 2]...They had no knowledge of me. And then all of a sudden, out of the blue, I got a phone call from [primary care service], asking me to go through there the following day” (Patient)*

GDPs felt that they had not been provided with information about the new service in a sufficiently timely way, despite the commissioning team sending letters and holding information meetings prior to implementation and providing briefings to the Local Dental Committee. Staff from referring practices tended to blame the commissioning team for failing to communicate adequately. It took several months for staff within the referring practices, the patients, the advanced service and the evaluation team to feedback to each other before a shared understanding around the service and its aims could begin to develop.

*“It’s five minutes down the road, the guy that did it [DWSPI], which is great. But it was just, we weren’t told he was there and then it got us into a tricky situation. So, in terms of the Department of Health… I’ve no real faith in them getting us the information on time” (practice manager).*

A lack of coherence around understanding new treatment pathways had led to confusion around *where* treatment would take place. In addition, patients lacked an understanding of the clinical reasons for referral and why they were chosen to be treated by a specific provider, in a particular setting. This suggests that patients may not take an active role in the referral process at initial GDP appointments. Discovering they have been referred to a new service may however prompt patients to ask new questions about the type of treatment they can expect.

*“I didn’t know if the referral clinic where I was sent to was going to just give me the same injection and try to get it out again. So, I rang up and I said will I be sedated, or anything like that? Because I didn’t know what was going to happen to me after having this referral.” (Patient*)

A lack of coherence around the early implementation of the advanced service was apparent particularly in the early months of the service becoming operational. For patients, this seemed to affect their overall impression of the quality of the service offered however it did not appear to be a barrier to patients’ attendance at their appointments and completion of treatment. The primary care oral surgeon however felt this lack of awareness from GDPs and patients subsequently affected the numbers of referrals received.

*“So, all the initial hard work has been done, setting up the practice and everything ... On the other hand, now I’m not having enough patients to do that. I need more patients” (Primary care advanced service provider).*

### Collective action between providers

Some of the issues around developing a shared sense of coherence between GDPs and patients were attributed to a lack of initial operational work to inform and facilitate referrals from general dental practice. Often, information regarding the aims of the new service was absent or was not cascaded within general practice teams. The surgeon working in the new service had concerns regarding the integration of referral management (and subsequent primary care diversion) into everyday practice. He felt that information and training of new associates in the referral system was insufficient, resulting in some GDPs, particularly new staff members, bypassing the referral management system and referring directly to secondary care consultants.

*“But what happens is most of the big practices, they have more dentists changing, the new dentists come”. Those people, they don’t understand the referral system, unless the practice principal sits with them and explains to them. But more understanding, more teaching for the dentists, “this is how the system is going to work, and it’s a quick service and you have a lot of advantages” (Primary care advanced service provider).*

The need for effective communication is important throughout the whole of the system network. Demand management is a complex process that requires strong clinical leadership against a backdrop of risk recognition and patient safety and experience. Commissioners have highlighted that clinical governance is crucial and it is important that primary care Level 2 providers have a recognised and experienced consultant providing clinical supervision.

*“I think some lessons learnt there about being absolutely clear who is providing that service, and if it is a dentist with enhanced skills, and it may well be, that we have them linked into consultants or advanced for clinical supervision” (Commissioner).*

Relational integration refers to the knowledge work carried out in order to build accountability and maintain confidence in a new set of practices and with other participants as a new intervention develops [30]. Communication between and within the skill set involved in making the referral management system work is vital to ensure cost benefits are balanced with clinical quality, service experience and benefits to the broader NHS network. For example, consultant triagers need to feel they have enough information about the primary care provider to select and divert appropriate patients out of the hospital system, according to the provider’s skill set. A meeting involving triagers and the primary care service provider took place during the first year of the new service becoming operational was found to be useful in facilitating communication between both parties to identify any clinical and safety issues, refining boundaries for referrals and encouraging two-way communication between consultant triagers and the advanced primary care service.

*“It can’t be faceless, to be quite honest. I mean, just from the initial meeting we had with the current level two provider, you got the impression there were certain things he’d be happy doing and certain things he wouldn't, and without that meeting you wouldn’t know. And then you’re wasting everybody’s time then, because if you send something that’s too complex the patient’s wasting time, they’re going for an additional consultation, they’re disappointed” (Consultant).*

### Comparison with other treatment experiences

Although patients could be initially confused about their treatment pathways, most patients found the prospect of being treated in a primary care setting acceptable. Typically, their focus tended to be on having symptoms alleviated in a timely manner and was indifferent regarding whether their treatment took place in hospital or in a local setting, particularly when they had little prior experience of referrals to hospital for dental treatment. They had confidence in the ability of the NHS to provide safe and appropriate treatment in a general sense and were unconcerned about how it was being provided.

*“No it doesn’t bother me, because the setting was just the same. The one in the hospital it’s perhaps a bigger room, but it’s still the same, obviously, you’re only sat in a dentist’s chair aren’t you?” (Patient)*

When patients had previous experience of dental treatment in a hospital setting, a comparative experience upon which to base what May et al. (2009) refers to as an *individual appraisal* was present*.* These patients had certain expectations based on previous history of hospital treatment upon which to draw their appraisal of the new service. For example, some patients, were concerned by the dearth of artefacts and clinical surroundings associated with a hospital setting, such as dedicated car parking and a sterile environment.

*“I have to say the professional care was absolutely fine …it was just that the general surroundings…I didn’t know where to park my car because it's on a main road. There was a pub across the road but I didn’t like the look of that so I just parked up a side street. But the treatment was absolutely fine. The dentist was lovely, the dental assistants were nice and all the rest of it” (Patient)*

However, as shown in the example above, patients tended to be satisfied with their treatment and many patients made positive comparisons with the hospital service. Patients were particularly positive about shorter waiting times for treatment and having a choice of out of hours appointment times at one particular site. Although some patients had arrived at their first appointment at the advanced primary care service expecting to have an initial consultation, followed by a different appointment for treatment (as per the conventional hospital pathway) they generally preferred to have consultation and treatment over with at the same appointment.

*“When I went in to see this guy in [primary care service], he said to me, they could speed up things so you don’t have so long to wait and do it in one go. Certainly, when I phoned the hospital they had said there’s at least a three-month waiting list and that’s just for a consultation” (Patient)*

## Discussion

The aim of this case study was to assess safety, quality, acceptability and implementation of a new primary care service of oral surgery. The primary care service was acceptable to patients, who appear to have an expectation of treatment in hospital only if they have previous experience of oral surgery in secondary care settings. Patients prefer the shorter waiting times for appointments and to be assessed and treated at the same appointment. They were generally happy with the staff and their clinical skills and like the convenience of being treated locally.

Audit data showed that the service received referrals at the appropriate levels of treatment complexity via the electronic referral management service and most (65% of patients included in the audit) received initial consultation and treatment at a single appointment. In comparison, patients attending hospital outpatient services for oral surgery typically have to make at least two visits; an initial consultation, followed by a further appointment for tooth extraction. This offers patients an attractive alternative to hospital care incorporating a reduction in time needed to be taken from work or usual routines and costs associated with travel to and from appointments. Complications following treatment were also low, with a small number of patients returning to their general practice for after care advice, which reelected the good level of satisfaction with the service reported by participants.

Staff at the new primary care oral surgery service were working slightly below available capacity. This suggests some patients who may have been suited to treatment in this setting were not being referred to the service. This may be due to implementation issues, such as some general practice staff initially being unaware of the primary care service or lacking in a coherent understanding of its objectives. On some occasions patients chased their own referrals, contacting practices and hospitals before discovering the existence of the primary care service in a serendipitous manner. This was attributed by GDPs to lack of information disseminated in a timely and effective way by commissioning teams. In addition, the primary care surgeon felt that new staff members in primary care practices were not updated with details of the local service reorganisation and dentists moving from an area where referral management was not in place would not be familiar with the service provision for oral surgery in the pilot site.

There were four complications experienced by patients who attended the primary care service and consented to have their dental records examined, which were not excessive when compared to complications experienced by patients attending hospital services. However, concerns were expressed by the commissioning team and consultants around the provision of effective clinical supervision for the surgeon carrying out treatment in the primary care service. In order to ensure consistency of care and the provision of a safe service, it is important than a named, senior consultant is appointed as clinical supervisor and that effective communication takes place between the primary care service and secondary care oral surgeons. This can ensure appropriate decision-making takes place regarding clinical boundaries for borderline level 2 and 3 levels of case complexity. This is also important to ensuring that patients receive an efficient service overall, in that they are not being asked to make unnecessary visits to different service providers and to attend more appointments than necessary.

With appropriate clinical governance arrangements and effective communication with secondary care in place, the new service can provide oral surgery services in a safe and efficient manner. The service is acceptable to patients, with shorter waiting times to treatment, fewer appointments and convenient locations being positive features. Care needs to be taken during the early implementation period to ensure general dental practices are sufficiently informed about changes to referral pathways and service provision. Thus, they can prepare staff to refer patients to the new service appropriately so all suitable patients can benefit from reduced waiting times and fewer appointments prior to surgery.

This case study sits within a wider body of work which aimed to develop and evaluate a new intervention for demand management, which combined electronic referral management with the introduction of a new primary care service. Findings from from this case study contribute to the overall research through exploring the implementation of a service that is part of this larger, complex intervention. Within the context of the RE-AIM framework [31] this study addresses the A (adoption) and I (implementation) for translating the results of research into practice and should be considered part of a series of four publications addressing this much wider body of research (the overall report, this case study, implementation of the referral management system across time, and a health economic evaluation).

The application of NPT [26, 32] in the analysis of qualitative data allows for the extraction of a more abstract level of ideas for implementation of complex interventions, which could apply to other contexts and research settings and links to the aforementioned series of papers. NPT is a theory that explains what people to do to accommodate change in practice, rather than what their attitudes are. The findings from this study found participants responses mapped onto three domains: coherence, collective action and reflexive monitoring. A fourth domain, cognitive participation was less relevant to participant’s responses. Cognitive participation describes the relational work people do to build a shared community around new practices. As this case study largely involved an established primary care dental team, possibly the necessary interpersonal relationships needed to sustain new ways of working were already established.

Issues related to the domain of coherence (the sense-making work individuals and groups do when relating to a set of practices) seemed to be caused by the lack of information passing between more disparate groups of participants (from commissioning teams to dental practices on to patients) and this manifested through barriers to establishing collective action, the operations groups and individuals need to carry out to successfully put in place new practices. Despite initial barriers, appraisal of the system was largely positive and mapped onto the construct of reflexive monitoring. This suggests that, ultimately groups of professionals were able to work together to provide a service that patients found acceptable, safe and satisfactory.

The limitations of the study are that it focuses on one service, in one medical specialty, in one geographical area and the findings may not be generalizable to other settings. Further research is needed to investigate if new primary care services set up to manage high demand secondary care outpatient services in other geographical areas, under different geopolitical contexts and for different specialties yield similar results.

## Conclusion

Provided there is robust communication between all professional groups around the introduction of the new service and its aims, and adequate clinical governance is put into place, patients without existing complex medical needs who require oral surgery can be treated safely and efficiently in a primary care setting. These findings offer a potential solution for the management of other high demand secondary care services with long waiting lists for treatment in secondary care, such as dermatology.

## Abbreviations

DNA: Did Not Attend (appointment)
DwSpI: Dentist with Special Interest
GDP: General Dental Practitioner (Primary Care)
NHS: National Health Service (United Kingdom)
NPT: Normalization Process Theory
QUIPP: Quality, Innovation, Productivity and Prevention
